# A Case Report of an Unusual Presentation of Epiglottic Cyst Causing Airway Obstruction in an Adult

**DOI:** 10.7759/cureus.35863

**Published:** 2023-03-07

**Authors:** Vishnu Varathan, Marina Mat Baki, Santhi Kalimuthu

**Affiliations:** 1 Otolaryngology, Universiti Kebangsaan Malaysia, Klang, MYS; 2 Otolaryngology, Universiti Kebangsaan Malaysia Medical Center, Kuala Lumpur, MYS; 3 Otolaryngology - Head and Neck Surgery, Hospital Tengku Ampuan Rahimah Klang, Klang, MYS

**Keywords:** acute airway obstruction, ent procedures, intubation complication, difficult airway management, epiglottic cyst

## Abstract

A laryngeal cyst is usually benign. The most common site origin of a cyst in the larynx is the lingual surface of the epiglottis. Epiglottic cysts are rare. Usually, it is asymptomatic in patients and can be treated conservatively if the size is small. Airway obstruction is very rare but could be life-threatening. If there is airway obstruction, the cyst should be removed immediately. Early detection and immediate management lead to favorable outcomes. A multidisciplinary-team approach with the ENT and anesthetic inputs are essential when dealing with the current condition. This case report highlights the management of such a patient who presented with airway obstruction due to an epiglottic cyst.

## Introduction

Epiglottic cysts are benign and usually occur on the lingual surface of the epiglottis. The occurrence is very rare and normally discovered in patients during acute infection. Usually, it is asymptomatic in patients, and epiglottic cysts have been treated conservatively if the size is small. Tuang et al. stated in an infant, they may cause sudden death, but in adults, they appear as a lumpy sensation and the clinician might miss it. They are mostly asymptomatic but if the size is large, it may obstruct the airway and can result in upper airway obstruction. If there is any sign of airway obstruction, the cyst should be removed immediately. Once the cyst is detected, it can be managed via endoscopic resection or marsupialization. Immediate management is preferred. A multidisciplinary-team approach between the anesthetic and ENT team will bring a great approach to treating patients with this condition [1-3]. Here we report a patient with an epiglottic cyst who had complete airway obstruction and describe the multi-disciplinary approach to manage his condition.

## Case presentation

**Figure 1:**
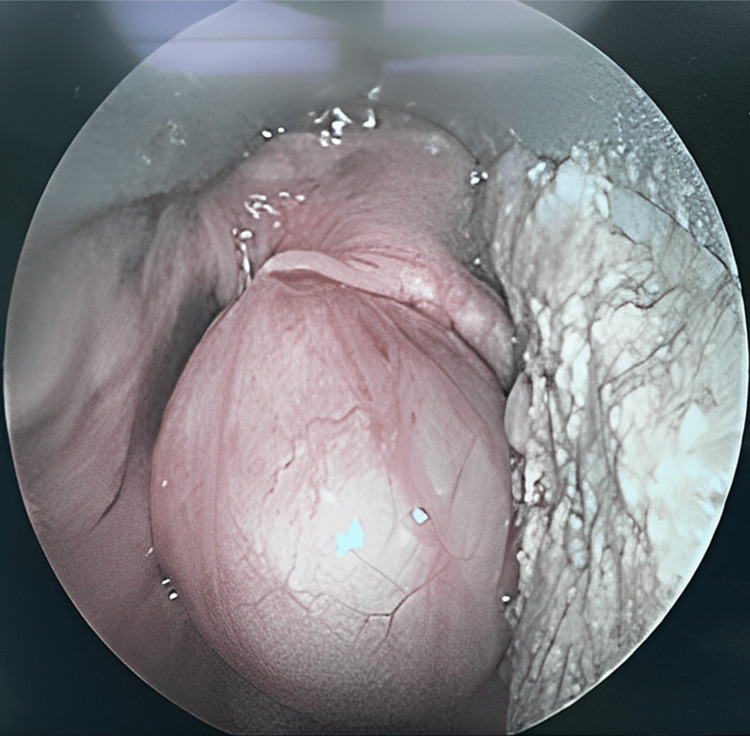
Direct laryngoscopic view of the epiglottic cyst covering the whole airway

**Figure 2:**
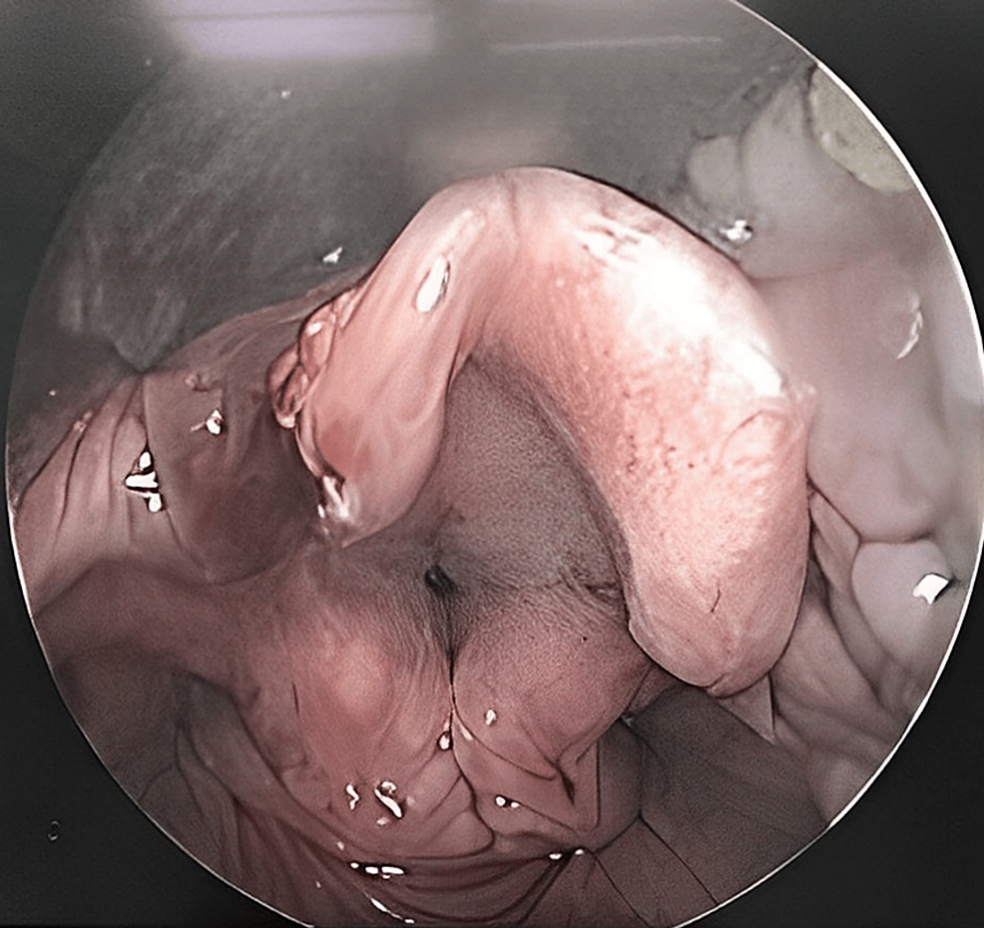
Postoperative image

We would like to present a case of a 40-year-old gentleman, a heavy smoker, who presented to the emergency department with a foreign body sensation in the throat for six months but worsening in symptoms for three weeks. The patient experienced intermittent shortness of breath lying flat, and often have to bend down to make himself relieve of the symptom. Otherwise, there was no history of stridor, dysphagia, tachypnea, or hoarseness. The patient was comfortable with an oxygen saturation of 98% to 100% under room air. On examination, there were no palpable neck masses, and the oral cavity was normal with no trismus. Flexible endoscopy revealed a round pedunculated mass arising from the left epiglottis obstructing the whole airway (Figure 1). The airway was secured by performing a tracheostomy under local anesthesia after multiple attempts of failed nasal intubation. This nasal intubation is done with standard ETT and IV routine induction. It was attempted because the patient was not cooperative lying supine due to the airway obstruction by the epiglottic cyst. However, during the awake fiberoptic nasal intubation, the patient was agitated and had a few episodes of desaturation to 60%. Subsequently, an excision of the epiglottic cyst under general anesthesia was done (Figure 2). A huge pedunculated cystic mass originated from the right aryepiglottic fold obstructing the whole airway seen via direct laryngoscope intraoperatively. Postoperative day 3, the tracheostomy tube was downsized from size 7.5 to 6 and was a spigot. After 24 hours, the patient was comfortable, had no stridor, saturating well on room air, not in respiratory distress with good voice modulations. The tracheostomy tube was decannulated prior to discharge.

## Discussion

The etiology of the epiglottis cyst still remains unknown. It commonly arises from the lingual surface of the epiglottic region. De Santo et al. divided laryngeal cysts into ductal and saccular types based on histological components. Based on our histopathological findings of the left epiglottic cyst, it was revealed as a ductal cyst. A newer system by Forte, et al, classifies the laryngeal cyst based on the extent and embryologic tissue of origin. In pediatrics, common presentation is stridor and respiratory distress, and adults usually present with foreign body sensation in the throat, and hoarseness, which seldom leads to respiratory distress. Common presentations in an adult will be a muffled voice, dysphagia, odynophagia, and biphasic stridor but there was no such presentation in our patient [1-3].

Normally, a lateral x-ray of the neck shows a soft tissue shadow around the epiglottic area and computed tomography would be the imaging of choice and useful tool for immediate confirmation of the diagnosis. In our patient, awake fiberoptic intubation was attempted because it is the gold standard for any difficult intraoral, and the patient was not exposed to induction agents and muscle relaxants. The advantage of awake fiberoptic intubation will prevent the patient to avoid post induction CICO (Can't intubate, Can't Oxygenate) situation. Besides, awake fiberoptic is performed because the upper airway can be examined before induction of the anesthesia which is helpful for diagnostic purposes and to predict how difficult the intubation would be [3]. However, when the laryngeal inlet could not be visualized on a flexible nasopharyngolaryngoscopy, awake fiberoptic intubation may cause “cork in the bottle” obstruction or laryngospasm in severe acute airway obstruction.

Treatment of epiglottic cysts depends on their size and severity of airway obstruction. Patients with an asymptomatic epiglottic cysts can be observed, larger cysts require surgery. One of them is marsuplisation or endoscopic excision of cysts. A few cases reported performing carbon dioxide laser during endoscopic surgery has been effective due to its good hemostatic effect [4,5].

Besides, the cyst can be aspirated via a long needle to reduce the size of the cyst using a 22-Gauge spinal needle which enables the cyst to shrink and enables the anesthesiology team to intubate using the Macintosh laryngoscope which is a safer way to avoid unnecessary tracheostomy. In some cases, the lateral pharyngectomy method is used especially in recurrent cases. In our case, despite the administration of muscle relaxation drugs, the patient was still not cooperative because he was having difficulty breathing due to an epiglottic cyst that totally obstructed the airway causing the patient to be more restless. However, manipulation of the larynx made, by lying side to side but failed. Using blades such as Miller blade or McCoy carries the risk of rupture of the cyst where the patient can have laryngospasm if there is any spillage of the cyst into the trachea which leads to cardiac arrest in the worst-case scenario and we proceeded with tracheostomy under local anesthesia. After the tracheostomy, the patient was commenced under general anesthesia, and marsuplization of the cyst was done via direct laryngoscopy. Studies show that supraglottic airway devices are useful, but it also has the risk of rupture [4,6].

Moreover, we can also use the curved or bend-needle trans-orally with the guidance of the flexible nasolaryngophayrngoscope to aspirate the cyst. By performing this, the size of the epiglottic cyst gets smaller and is able to relieve the airway till OT calls and to ease the intubation. This can be done in clinical settings and when the patient is awake. The patient will be sitting upright position where lidocaine 2% about 1 to 2 mL injected via scope and flushed to the laryngeal inlet. The patient will cough and subsequently patient will feel numbness over the laryngeal region and chances of bleeding, hematoma, and aspiration which limits access. In our case, the epiglottic cyst is pedunculated and obscuring the airway, and the patient was in impending airway obstruction with intermittent shortness of breath. We anticipated the risk of aspiration of cystic content into the airway which can cause irritation and further compromise the airway. The best way is to perform aspiration in a controlled setting with the anesthetic team [7-9].

## Conclusions

An epiglottic cyst can be one of the causes of airway obstruction. Treatment of epiglottic cysts depends on the size and severity of airway obstruction. Patients with an asymptomatic epiglottic cyst can be observed, larger cysts require surgery. Early diagnosis can avoid unnecessary tracheostomy. Maintaining the airway and preventing the rupture of cysts play a crucial role in the airway management of epiglottic cysts.
